# CRISPRStudio: A User-Friendly Software for Rapid CRISPR Array Visualization

**DOI:** 10.3390/v10110602

**Published:** 2018-11-01

**Authors:** Moïra B. Dion, Simon J. Labrie, Shiraz A. Shah, Sylvain Moineau

**Affiliations:** 1 Département de Biochimie, de Microbiologie, et de Bio-informatique, Faculté des Sciences et de Génie, Université Laval, Québec City, QC G1V 0A6, Canada; moira.dion.1@ulaval.ca; 2 Groupe de Recherche en Écologie Buccale, Faculté de Médecine Dentaire, Université Laval, Québec City, QC G1V 0A6, Canada; 3 SyntBioLab Inc., 4820 rue de la Pascaline, Lévis, QC G6W 0L9, Canada; simon.labrie@syntbiolab.com; 4 COPSAC, Copenhagen Prospective Studies on Asthma in Childhood, Herlev and Gentofte Hospital, University of Copenhagen, Ledreborg Alle 34, 2820 Gentofte, Denmark; shiraz.shah@dbac.dk

**Keywords:** CRISPR, typing, software, visualization, viral origin, phage

## Abstract

The CRISPR-Cas system biologically serves as an adaptive defense mechanism against phages. However, there is growing interest in exploiting the hypervariable nature of the CRISPR locus, often of viral origin, for microbial typing and tracking. Moreover, the spacer content of any given strain provides a phage resistance profile. Large-scale CRISPR typing studies require an efficient method for showcasing CRISPR array similarities across multiple isolates. Historically, CRISPR arrays found in microbes have been represented by colored shapes based on nucleotide sequence identity and, while this approach is now routinely used, only scarce computational resources are available to automate the process, making it very time-consuming for large datasets. To alleviate this tedious task, we introduce CRISPRStudio, a command-line tool developed to accelerate CRISPR analysis and standardize the preparation of CRISPR array figures. It first compares nucleotide spacer sequences present in a dataset and then clusters them based on sequence similarity to assign a meaningful representative color. CRISPRStudio offers versatility to suit different biological contexts by including options such as automatic sorting of CRISPR loci and highlighting of shared spacers, while remaining fast and user-friendly.

## 1. Introduction

Of all the applications and technologies developed around CRISPR-Cas systems, strain typing is arguably the oldest one, as it capitalizes on the ability of the system to acquire new DNA fragments [1]. Initially discovered as an unusual nucleotide arrangement in the *Escherichia coli* genome [2,3] and later as a defense mechanism [4], CRISPR (Clustered Regularly Interspaced Short Palindromic Repeats) loci, together with their *cas* (CRISPR-associated) genes, form the so-called CRISPR-Cas systems. These microbial systems provide resistance against invasion of foreign DNA elements, such as phage genomes and plasmids [4,5], by first incorporating short DNA sequences called spacers [6] which originate from the foreign DNA element, into the CRISPR array. In the case of phage infections, the CRISPR-Cas machinery will then use these spacers of viral origin as a memory to produce crRNAs that will specifically target subsequent infections by phage genomes carrying the exact (proto) spacer sequence. Such targeted invading viral sequences will then be cleaved, thereby blocking the phage infection process [5]. Because the integration of spacers is mostly polarized to the 5′ end of the locus [6] in a chronological manner, CRISPR arrays often behave as a molecular archive of previous phage infections or plasmid encounters [7]. This feature, combined with the evolving structure of the CRISPR locus, results in a hypervariable DNA region that provides information on strain origin, evolutionary path and even phage resistance/sensitivity profiles. Thus, CRISPR typing has been used to identify and greatly improve strain typing resolution for a variety of bacterial species in clinical, epidemiological, environmental and industrial settings [8,9,10,11,12,13,14,15,16,17,18,19,20,21,22,23].

Usually, CRISPR typing analyses begin by comparing the spacer content (number of spacers and nucleotide sequences) of different bacterial isolates to organize and cluster them based on their spacer content similarity. Then, for reporting purposes, the CRISPR arrays are graphically represented with colored shapes, making it easy to visualize similarities and differences across microbial strains. Although this representation is now commonly used to facilitate comparison and analyses [17,18,19,20,21,22,23,24,25,26,27,28,29], the development of software to support such analyses remained unexplored until recently when the first automated visualization program, called CRISPRviz, was published [30]. This program is a web-based visualization tool that allows rapid conversion of nucleotide sequences to colored shapes, for more intuitive strain comparison. Although CRISPRviz alleviates considerably the repetitive nature of visualizing spacers with colored shapes, it has some limitations. First, CRISPRviz relies on minCED [31] for spacer and repeat extraction and while it is a rapid program for CRISPR identification, it does not recognize the array orientation. As such, the user must manually verify the reverse complement sequence in the output interface, which may become tedious in large-scale studies. Second, the system for color-coding spacers, which consists of transforming nucleotides to integers and then to a RGB (red, green, blue) value, causes nearly identical spacers to be represented by completely different colors. This may be relevant in some bacterial species, where a single nucleotide difference between a spacer sequence and its protospacer equivalent in the phage genome can alter the cell’s resistance [32]. In other bacterial species however, for example *E. coli*, it has been shown that the nucleotide sequence matching requirement for a spacer to efficiently block phage infection and cleave nucleic acids, also known as the seed region, is much shorter [33,34]. Hence, nucleotide sequence comparisons of spacers should be tailored to their biological context and allow a customizable number of mismatches.

To facilitate and expedite the graphical representation of CRISPR loci as well as CRISPR typing analyses, we introduce CRISPRStudio, a software for generating presentation-ready figures for CRISPR typing studies. CRISPRStudio automatically converts spacers to a color-coded figure by first aligning and clustering spacers based on a predefined similarity cut-off, and then assigning the same color code to spacers clustered together. CRISPRStudio offers a substantial improvement in efficiency and accuracy when preparing figures for analyses and publications. The color-coded visualization is an intuitive method to identify strains with the same phage infection profile and conserved spacers among evolutionary distant microbial species, suggestive of infections by broad host range phages.

## 2. Materials and Methods

**Figure 1:**
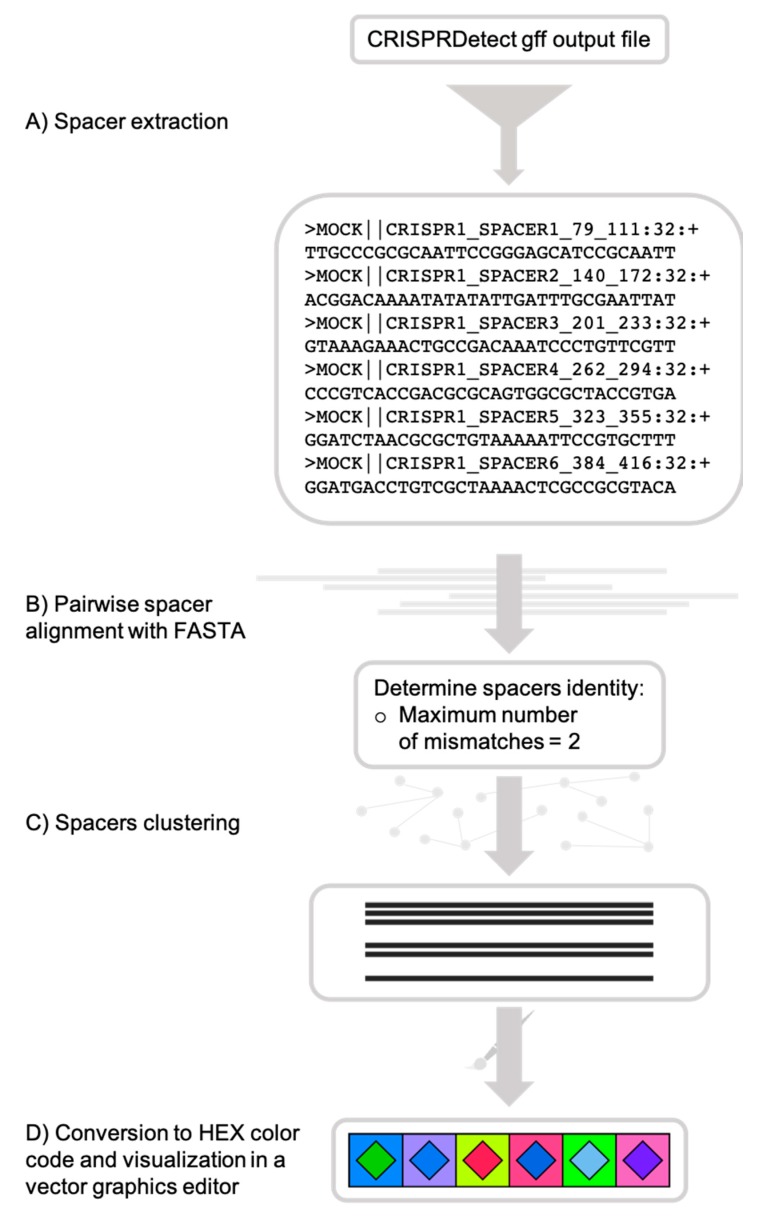
CRISPRStudio workflow scheme. (**A**) Spacer sequences are first extracted from the CRISPRDetect gff output file, (**B**) then aligned with fasta36, and (**C**) clustered, based on the mismatch cut-off. (**D**) Each cluster is assigned a two-color code and an SVG (scalable vector graphics) file is produced for visualization.

CRISPRStudio is a Python script and should run on any Unix operating system equipped with Python 3.6.x or older, fasta36 [35] and the following Python packages: SciPy, pandas, NumPy and scikit-bio. An automatic installation of all the dependencies is provided upon download of the program from the GitHub repository [36]. For technical support, a built-in help menu is available when executing the program and command examples are also demonstrated on the GitHub repository. Figure 1 summarizes the workflow to obtain color-coded figures of CRISPR loci.

### 2.1. CRISPR Mining and Spacer Extraction

CRISPRs must first be retrieved from raw DNA sequences. CRISPRDetect [37] is arguably the most accurate and user-friendly software currently available for CRISPR mining. Using the CRISPRDirection algorithm, it can predict the orientation of CRISPR arrays, as well as the CRISPR-Cas types. The prediction of the array orientation is particularly important when comparing spacer content and position across different microbial strains because all the spacers should be displayed in the same orientation to allow proper comparison. For smaller datasets, CRISPRDetect may even be used from its interactive web interface. Consequently, we implemented the workflow around the general feature format standard (version 3–gff3). By default, a gff3 file is generated when executing CRISPRDetect. Each CRISPR array is defined by the feature type “repeat_region” that encompasses the direct repeats and spacers. For each array, CRISPRStudio extracts the sequences corresponding to the CRISPR spacers (Feature type = “binding_site” or “Spacer”). The spacers are then identified with the sequence name (field #1–seqname), CRISPR locus number, spacer number (sequentially attributed within the array), start (field #4) and end (field #5) positions, sequence length (field #6) and orientation (field #7). Spacers for the complete dataset are saved in one file using the FASTA format. Although it is not part of our pipeline, this file is well formatted for homology searches with protospacers sequences in phage genomes.

### 2.2. Spacers Comparison and Conversion to HEX Color Code

To assign a color for visualization and analysis, spacers must be aligned to determine which ones are identical and, as such, be assigned the same colors. Due to its high sensitivity, fasta36 is used to perform the local alignments. Two spacers are considered identical when they have an adjusted number of mismatches less than or equal to a threshold defined by the user, which is set to two by default. The adjusted number of nucleotide mismatches is the number of mismatches across the full length of the query sequence and is calculated using the following formula:

Adjusted number of nucleotide mismatches = query spacer length − (alignment length − number of mismatches − number of gaps)(1)

A default cut-off of two mismatches ensures a strict pairwise alignment while allowing for occasional sequencing errors for each spacer. Although it is recommended to maintain a low mismatch cut-off, this value can be modified while running the program. Next, the pairwise similar spacers are converted to clusters of similar spacers by transitivity, that is if spacer A is similar to spacer B and spacer B is similar to spacer C, then spacer A, B and C form a cluster. Each cluster contains at least one spacer and is assigned two random three-digit hexadecimal (HEX) codes corresponding to the square and the diamond colors, respectively.

### 2.3. Default CRISPR Array Clustering

CRISPRStudio will by default display the strains based on a guide tree generated after hierarchical clustering. The program first computes a strain pairwise comparison of the number of shared spacers and converts it to a symmetrical dissimilarity matrix following the Bray–Curtis metrics. The tree is then built using the UPGMA functionality implemented in the SciPy python module, a hierarchical clustering approach to reflect the structure of a dissimilarity matrix. Finally, the leaf order of the guide tree is used to sort the isolates in the final display step.

### 2.4. Visualization in a Vector Graphics Editor

Lastly, CRISPRStudio writes a file in Scalable Vector Graphics (SVG) format which can be opened in graphics editing software, such as Adobe Illustrator or Inkscape. Each spacer is displayed as a colored square with a colored diamond in its center. CRISPR loci are shown on individual rows in a 5′ to 3′ orientation. If a sample carries multiple CRISPR loci, they are split into different columns. Every CRISPR locus is lined up along the right side, followed by the sample name at the rightmost end of the row.

### 2.5. Performance and Additional Feature Demonstrations

To test the program speed, we used a previously well-described dataset from *Salmonella*, consisting of 206 draft genomes publicly available [27]. The program was executed on a Mac running OS X on four CPUs. A subset of 74 strains were selected to exemplify the CRISPR array clustering feature. To compare our color-coding approach with that of CRISPRviz, we used the type I-E CRISPR 1 array of *E. coli* strain K12 NEB 5-alpha (NZ_CP017100.1). The CRISPR 1 loci of seven strains of *Streptococcus thermophilus* were used to demonstrate the graying out feature. The 74 *Salmonella* strains, *E. coli* and *S. thermophilus* datasets are described in Table S1 and are available as Supplementary Data and on the GitHub repository as test files.

## 3. Results

### 3.1. Speed Evaluation

CRISPRStudio was developed to accelerate and standardize the presentation and analysis of CRISPR profiles of microbial strains. The 206 *Salmonella* isolates carried a total of 4705 spacers, which were displayed in under five minutes. The limiting factor in the pipeline is the alignment step with fasta36. Its processing time is proportional to the total number of spacers in the dataset.

### 3.2. Color-Coded Improved Visualization

**Figure 2:**
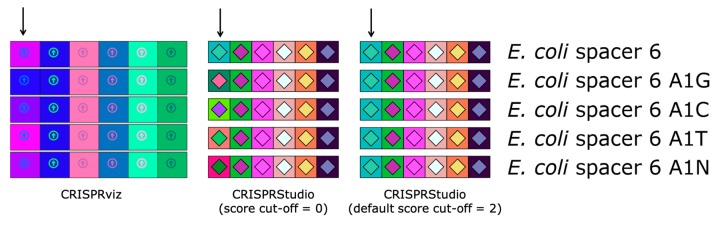
Comparison of color-coding systems of CRISPRviz and CRISPRStudio. Each row represents the same array with the exception of the sixth spacer (marked with an arrow) for which we changed the first nucleotide (A to G, C, T or N). CRISPRviz displays spacers with the same color only when they have identical sequences. This results in spacers with only one mismatch having different colors depending on the nucleotide change. CRISPRStudio offers a customizable number of mismatches. When the score cut-off is set to zero, only spacer pairs without mismatch will have the same color. By default, the score cut-off is set to 2, which allows for mutations and/or sequencing errors, without changing the spacers colors.

Since new spacers are mostly added at the 5′-end of the CRISPR array during the viral adaptation stage, while spacer deletion has been reported to occur in the internal part of the CRISPR array, it is commonly accepted that the most ancient and conserved spacers are found at the 3′-end of the array [12,38]. By aligning CRISPR loci on the right (in the 5′–3′ orientation), spacers at the 3′-end, which are more likely to be shared among microbial strains of the dataset, will also share their vertical position, making similar spacers between samples easy to identify. Another improvement with our proposed color-coding method is the versatility it offers for comparing spacers. By default, two spacers will have the same color if their pairwise alignment shows at most 2 mismatches. This value can be modified by adding an optional argument when executing the program, so that spacer comparison becomes more stringent (score cut-off = 0) or more permissive (score cut-off > 2). This adjustment is not available with CRISPRviz, since the color-coding system consists of transforming nucleotide sequences to integers with a formula that does not allow different degrees of stringency. To illustrate this improvement, we compared the CRISPRviz output with that of our current program using the CRISPR 1 array from *E. coli* strain K12 (Figure 2). We modified the nucleotide on the first position of the sixth spacer of the array (A to G, C, T or N). With CRISPRviz, the degree of change in color varies according to the change in nucleotide: when A becomes N, the color looks more similar compared to when A becomes G. With CRISPRStudio, the colors either change completely with a score cut-off of 0 or remain the same with the default value of 2.

By using the number of mismatches as sole criterion for similarity, CRISPRStudio does not discriminate how these mismatches occurred. In this biological context, mismatches in spacers sequences can arise from mutations over time in the CRISPR locus, imperfect splitting of spacers and repeats from CRISPRDetect, sequencing errors or different acquisition events. The latter signifies that two microbial strains would have acquired different spacers from nearly identical positions in the phage genome (e.g., offset by one nucleotide). Considering that the likelihood of this event remains unknown, depends on the bacteria and that the vast majority of spacers have no homology with currently sequenced phage genomes to verify such event, we did not implement additional parameters to distinguish diverged spacers from different acquisition events. As such, depending on the mismatch cut-off, two spacers originating from independent acquisition events could be considered similar and be represented by the same color combination. Still, CRISPRStudio provides a FASTA file containing all the spacers sequences that can be used to do a similarity search with phage genomes or databases and manually identify different acquisition events.

### 3.3. Customized Sorting and Editing

**Figure 3:**
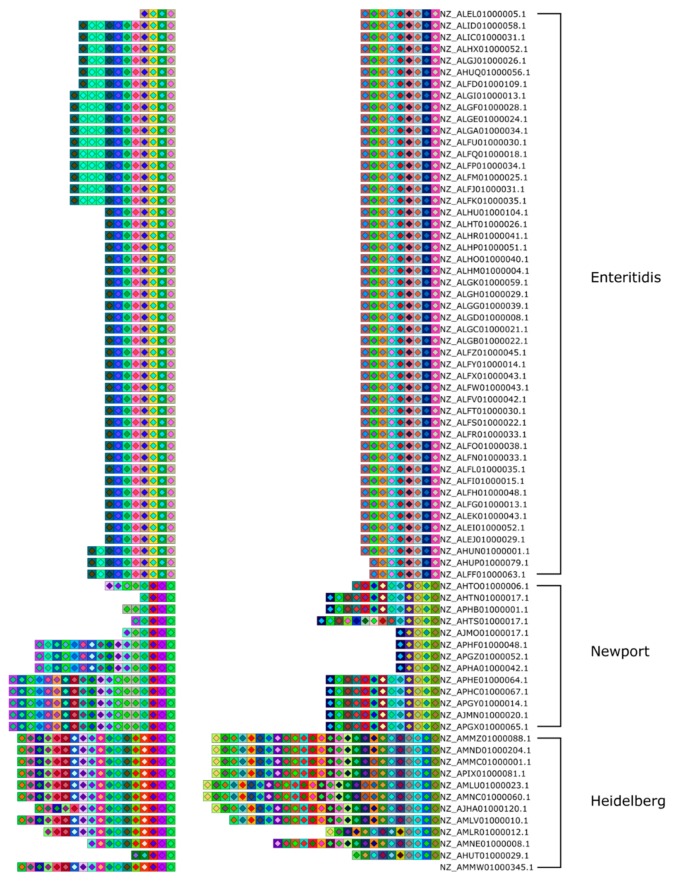
Array clustering method implemented in CRISPRStudio. A set of 74 *Salmonella* strains are displayed here in clusters of similar arrays. CRISPR 1 and CRISPR 2 are shown from right to left. The color-coding easily distinguishes the three major groups following the clustering, which correspond to different serotypes.

**Figure 4:**
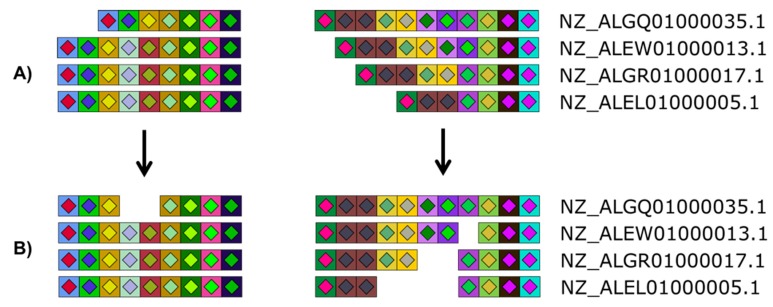
Versatility offered by CRISPRStudio. Vector graphics output allows for easy manual editing. Here, four *Salmonella* strains are displayed with their two CRISPR loci split in two columns. White spaces were added to the original figure (**A**) so that homologous spacers are vertically aligned (**B**).

By default, CRISPRStudio will display the samples/isolates based on a guide tree. The tree is built according to a dissimilarity matrix, which measures the number of shared spacers between each isolate. The isolates are then sorted based on the leaf order of the guide tree, which results in clusters of similar strains in the SVG file (Figure 3). The clustering automation adds great value to the program, by pruning the manual work involved in figure preparation which speeds up the process. Shared spacers, indicative of common phage infection events, are also readily identified. To suit other research settings, CRISPRStudio also offers to sort the samples based on a custom list. With that option, samples could be sorted by their sampling time, sampling location, species, or other relevant variables to the study. Furthermore, creating an SVG file makes it easy to adjust figures as required. White spaces may be added manually in the graphics editing software to extend loci so that homologous spacers are vertically aligned (Figure 4), and because the colored shapes are vectors, they may be scaled without loss of resolution.

### 3.4. Manual Inspection

**Figure 5:**
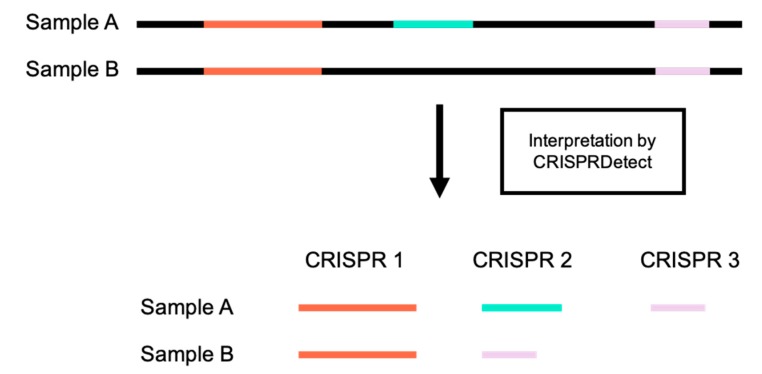
CRISPR loci misnumbering. CRISPR loci are given inconsistent numbers by CRISPRDetect in the cases where sequences from two samples do not contain all of the same CRISPR loci.

Although CRISPRStudio automates the process of CRISPR presentation, as a precautionary measure, the FASTA file generated by extracting spacers from nucleotide sequences should still be manually checked for errors before carrying out the rest of the commands. Firstly, CRISPRDetect may have mixed up repeats and spacers, which causes them to be incorrectly separated from each other. This happens when sequences have too many unknown nucleotides or mutations near the beginning or the end of the repeat. Such an incorrect spacer is easily noticeable in the FASTA file because it will be much longer than its expected length (most often one extra repeat and spacer). CRISPRStudio issues a warning when a spacer is 1.5× longer or shorter than the average spacer length of the dataset. The user may then manually fix the gff file or modify the FASTA file generated and restart the analysis. For low coverage sequences, CRISPRDetect may also split one CRISPR locus into two or more loci if too many unknown nucleotides are present. This can be fixed by manually changing the CRISPR locus id and spacer id in the FASTA file. An error of this kind may also be caught in the CRISPRDetect output text file by comparing the left and right flanking sequences with the spacers and repeats of other CRISPR loci originating from the same sample. Two loci should be merged when the flanking sequences of one locus match the repeats and spacers of the other locus. Finally, there can be an issue when labeling the CRISPR locus number if the dataset contains isolates with a variable number of CRISPR loci (Figure 5). Therefore, CRISPR locus numbering must be closely examined and manually adjusted to confirm its accuracy. To support the user in this process, we provided an option where the spacer length (numeric value) is added over its grayed out shape in the final display. When microbial genomes harbor more than one CRISPR locus, spacer size is indeed a way to differentiate loci that belong to different CRISPR-Cas systems. We recommend using this option only for exploratory analysis and not for final figure preparation.

### 3.5. Appending a Pre-Existing Dataset

Because CRISPRStudio assigns random colors to spacers each time the program is executed, we have added an optional parameter to keep the same two colors for each cluster when rerunning the program using the same dataset or the same dataset with new appended sequences. When new sequences are added, they are aligned and clustered with the entire dataset and random colors are assigned only to new clusters, assuming new clusters are formed.

### 3.6. Graying Out Spacers

**Figure 6:**
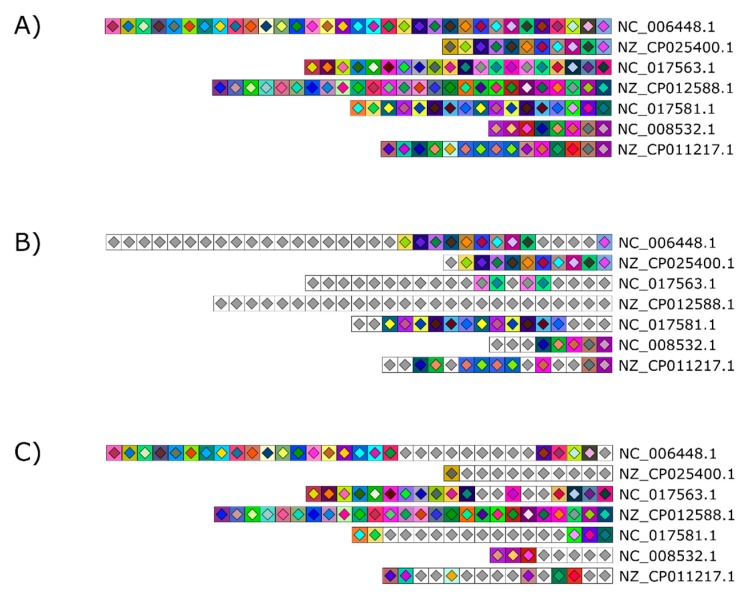
Optional graying out to easily mark similar and unique spacers across and within samples. (**A**) The CRISPR 1 loci of seven *S. thermophilus* strains were used to illustrate this feature. Two options are available: (**B**) Unique spacers present only once in the figure are grayed out to highlight similar spacers across the dataset or (**C**) similar spacers are grayed out to highlight unique colored spacers. Grayed out spacers all bear the same symbol consisting of a white square with a light gray diamond.

When reporting highly similar CRISPR loci across samples, color coding spacers makes figures self-evident. However, in the case of very dissimilar CRISPR loci, displaying every unique spacer with its individual two-color code becomes less meaningful. The multitude of colors makes it more difficult to quickly identify relevant spacer information. Conversely, highlighting only unique spacers may be relevant to show diversity or to focus on spacer content differences between samples. Thus, we added the option to gray out unique spacers or similar spacers. In the former case, only spacers present at least twice remain colored, while unique spacers all have the same white square and light gray diamond (Figure 6B). In the latter, similar spacers are grayed out and only unique spacers remain colored (Figure 6C). Such representation of CRISPR profiles allows for rapid identification of identical spacers in various strains and may quickly hint at evolutionary relationships, at genetic exchanges or points to past invasions by similar phages or other foreign genetic elements.

We developed CRISPRStudio to address speed and accuracy limitations in the generation of color-coded CRISPR loci figures as well as to simplify analysis and comparison of CRISPR profiles. Starting from a robust CRISPR mining output, our program is designed to compare spacers, cluster them based on their sequence homology and assign meaningful colors. The optional features allow users to rapidly customize figures, as needed, with minimal additional software requirements, for ease of use. Together, these results show the versatility of CRISPRStudio and its potential to support and greatly accelerate CRISPR array visualization in large-scale CRISPR typing and phage infection chronology studies.

## Supplementary Materials

The following are available online at http://www.mdpi.com/1999-4915/10/11/602/s1. Table S1: Description of the test files.

## References

[1] Lemay M.L., Horvath P., Moineau S. (2017). The CRISPR-Cas app goes viral. Curr. Opin. Microbiol..

[2] Ishino Y., Shinagawa H., Makino K., Amemura M., Nakata A. (1987). Nucleotide sequence of the *iap* gene, responsible for alkaline phosphatase isozyme conversion in *Escherichia coli*, and identification of the gene product. J. Bacteriol..

[3] Nakata A., Amemura M., Makino K. (1989). Unusual nucleotide arrangement with repeated sequences in the *Escherichia coli* K-12 chromosome. J. Bacteriol..

[4] Barrangou R., Fremaux C., Deveau H., Richards M., Boyaval P., Moineau S.A., Romero D., Horvath P. (2007). CRISPR provides acquired resistance against viruses in prokaryotes. Science.

[5] Garneau J.E., Dupuis M.È., Villion M., Romero D.A., Barrangou R., Boyaval P., Fremaux C., Horvath P., Magadán A.H., Moineau S. (2010). The CRISPR/Cas bacterial immune system cleaves bacteriophage and plasmid DNA. Nature.

[6] Nuñez J.K., Lee A.S.Y., Engelman A., Doudna J.A. (2015). Integrase-mediated spacer acquisition during CRISPR–Cas adaptive immunity. Nature.

[7] Barrangou R., Horvath P. (2012). CRISPR: New horizons in phage resistance and strain identification. Annu. Rev. Food Sci. Technol..

[8] Sun H., Li Y., Shi X., Lin Y., Qiu Y., Zhang J., Liu Y., Jiang M., Zhang Z., Chen Q. (2015). Association of CRISPR/Cas evolution with *Vibrio parahaemolyticus* virulence factors and genotypes. Foodborne Pathog. Dis..

[9] Bangpanwimon K., Sottisuporn J., Mittraparp-Arthorn P., Ueaphatthanaphanich W., Rattanasupar A., Pourcel C., Vuddhakul V. (2017). CRISPR-like sequences in *Helicobacter pylori* and application in genotyping. Gut Pathog..

[10] Ogrodzki P., Forsythe S.J. (2017). DNA-sequence based typing of the *Cronobacter genus* using MLST, CRISPR-Cas array and capsular profiling. Front. Microbiol..

[11] Shi L., Yang G., Zhang Z., Xia L., Liang Y., Tan H., He J., Xu J., Song Z., Li W. (2018). Reemergence of human plague in Yunnan, China in 2016. PLoS ONE.

[12] Horvath P., Romero D.A., Coûté-Monvoisin A.C., Richards M., Deveau H., Moineau S., Boyaval P., Fremaux C., Barrangou R. (2008). Diversity, activity, and evolution of CRISPR loci in *Streptococcus thermophilus*. J. Bacteriol..

[13] De Cardénas I., Fernández-Garayzábal J.F., de la Cruz M.L., Domímguez L., Ugarte-Ruiz M., Gómez-Barrero S. (2015). Efficacy of a typing scheme for *Campylobacter* based on the combination of true and questionable CRISPR. J. Microbiol. Methods.

[14] Van Belkum A., Soriaga L.B., LaFave M.C., Akella S., Veyrieras J., Barbu E.M., Shortridge D., Blanc B., Hannum G., Zambardi G. (2015). Phylogenetic distribution of CRISPR-Cas systems in antibiotic-resistant *Pseudomonas aeruginosa*. MBio.

[15] Zheng P.X., Chan Y.C., Chiou C.S., Chiang-Ni C., Wang S.Y., Tsai P.J., Chuang W.J., Lin Y.S., Liu C.C., Wu J.J. (2015). Clustered regularly interspaced short palindromic repeats are emm type-specific in highly prevalent group A Streptococci. PLoS ONE.

[16] Du Plessis M., Wolter N., Allam M., de Gouveia L., Moosa F., Ntshoe G., Blumberg L., Cohen C., Smith M., Mutevedzi P. (2017). Molecular characterization of *Corynebacterium diphtheriae* outbreak isolates, South Africa, March–June 2015. Emerg. Infect. Dis..

[17] Briner A.E., Barrangou R. (2014). *Lactobacillus buchneri* genotyping on the basis of clustered regularly interspaced short palindromic repeat (CRISPR) locus diversity. Appl. Environ. Microbiol..

[18] Hidalgo-Cantabrana C., Crawley A.B., Sanchez B., Barrangou R. (2017). Characterization and exploitation of CRISPR loci in *Bifidobacterium longum*. Front. Microbiol..

[19] Beauruelle C., Pastuszka A., Mereghetti L., Lanotte P. (2018). Group B *Streptococcus* vaginal carriage in pregnant women as deciphered by clustered regularly interspaced short palindromic repeat analysis. J. Clin. Microbiol..

[20] Lier C., Baticle E., Horvath P., Haguenoer E., Valentin A.S., Glaser P., Mereghetti L., Lanotte P. (2015). Analysis of the type II-A CRISPR-Cas system of *Streptococcus agalactiae* reveals distinctive features according to genetic lineages. Front. Genet..

[21] Andersen J.M., Shoup M., Robinson C., Britton R., Olsen K.E.P., Barrangou R. (2016). CRISPR Diversity and microevolution in *Clostridium difficile*. Genome Biol. Evol..

[22] Ogrodzki P., Forsythe S.J. (2016). CRISPR–Cas loci profiling of *Cronobacter sakazakii* pathovars. Future Microbiol..

[23] Tomida J., Morita Y., Shibayama K., Kikuchi K., Sawa T., Akaike T., Kawamura Y. (2017). Diversity and microevolution of CRISPR loci in *Helicobacter cinaedi*. PLoS ONE.

[24] Rauch H.E., Vosik D., Kariyawasam S., M’ikanatha N., Shariat N.W. (2018). Prevalence of group I *Salmonella* Kentucky in domestic food animals from Pennsylvania and overlap with human clinical CRISPR sequence types. Zoonoses Public Health.

[25] Xie X., Hu Y., Xu Y., Yin K., Li Y., Chen Y., Xia J. (2017). Genetic analysis of *Salmonella enterica* serovar Gallinarum biovar Pullorum based on characterization and evolution of CRISPR sequence. Vet. Microbiol..

[26] Shariat N., Dimarzio M.J., Yin S., Dettinger L., Sandt C.H., Lute J.R., Barrangou R., Dudley E.G. (2013). The combination of CRISPR-MVLST and PFGE provides increased discriminatory power for differentiating human clinical isolates of *Salmonella enterica* subsp. *enterica* serovar Enteritidis. Food Microbiol..

[27] Shariat N., Timme R.E., Pettengill J.B., Barrangou R., Dudley E.G. (2015). Characterization and evolution of *Salmonella* CRISPR-Cas systems. Microbiology.

[28] Almeida F., Medeiros M.I.C., Rodrigues D.D.P., Allard M.W., Falcão J.P. (2017). Molecular characterization of *Salmonella* Typhimurium isolated in Brazil by CRISPR-MVLST. J. Microbiol. Methods.

[29] Zheng H., Hu Y., Li Q., Tao J., Cai Y., Wang Y., Li J., Zhou Z., Pan Z., Jiao X. (2017). Subtyping *Salmonella enterica* serovar Derby with multilocus sequence typing (MLST) and clustered regularly interspaced short palindromic repeats (CRISPRs). Food Control.

[30] Nethery M.A., Barrangou R. (2018). CRISPR Visualizer: Rapid identification and visualization of CRISPR loci via an automated high-throughput processing pipeline. RNA Biol..

[31] Mining CRISPRs in Environmental Datasets. https://github.com/ctSkennerton/minced.

[32] Deveau H., Barrangou R., Garneau J.E., Labonté J., Fremaux C., Boyaval P., Romero D.A., Horvath P., Moineau S. (2008). Phage Response to CRISPR-Encoded Resistance in Streptococcus thermophilus. J. Bacteriol..

[33] Semenova E., Jore M.M., Datsenko K.A., Semenova A., Westra E.R. (2011). Interference by clustered regularly interspaced short palindromic repeat (CRISPR) RNA is governed by a seed sequence. Proc. Natl. Acad. Sci. USA.

[34] Sapranauskas R., Gasiunas G., Fremaux C., Barrangou R., Horvath P., Siksnys V. (2011). The *Streptococcus thermophilus* CRISPR-Cas system provides immunity in *Escherichia coli*. Nucleic Acids Res..

[35] Pearson W.R., Lipman D.J. (1988). Improved tools for biological sequence comparison. Proc. Natl. Acad. Sci. USA.

[36] CRISPRStudio. https://github.com/moineaulab/CRISPRStudio.

[37] Biswas A., Staals R.H.J., Morales S.E., Fineran P.C., Brown C.M. (2016). CRISPRDetect: A flexible algorithm to define CRISPR arrays. BMC Genom..

[38] Held N.L., Herrera A., Quiroz H.C., Whitaker R.J. (2010). CRISPR associated diversity within a population of *Sulfolobus islandicus*. PLoS ONE.

