# Supplementary material for: CRISPRStudio: A User-Friendly Software for Rapid CRISPR Array Visualization
Source: Viruses. 2018 Nov 1;10(11):602. doi: 10.3390/v10110602 (PMC6267562; doi:10.3390/v10110602)
Supplement: Supplementary file 1 [file viruses-10-00602-s001.zip › 2-viruses-378352-supplrmentary/resubmission/CRISPRStudio_supp_mat/Supplementary Materials.docx]

**Supplementary Materials**

**CRISPRStudio Installation**

CRISPRStudio runs on any Unix operating system equipped with the software requirements mentioned below. If your computer runs Windows 10, you can also install the Ubuntu terminal.

The program is available for download on GitHub:

<https://github.com/moineaulab/CRISPRStudio>

First clone the git on your computer by clicking on “Clone or download” → “Download ZIP”

Automatic installation

Open your terminal and go to the CRISPRStudio-master directory:

cd path/to/CRISPRStudio-master

The “install script” should install everything as long as a python3 and pip are already installed. To execute the script:

chmod u+x Install.sh

./Install.sh

In case of errors, you can perform a manual installation or raise an issue on the GitHub page. See the troubleshooting section for installation tips.

Manual installation

fasta36 can be downloaded at <http://faculty.virginia.edu/wrpearson/fasta/CURRENT/>

Help with fasta36 installation can be found here (follow step 1 to 3): <https://fasta.bioch.virginia.edu/fasta_www2/fasta_down.shtml>

Make sure you move the fasta36 file under the “CRISPRStudio-master” → “tools” directory

Finally, download the python modules from your terminal:

pip3 install scipy numpy scikit-bio pandas

**Troubleshooting**

1. Incompatibility

There seems to be incompatibility issues with scikit-bio and more recent versions of python3. Installation should work up to python 3.6.x, but our test was unsuccessful with 3.7.x. Check your python version by running:

python --version

If your python version is too recent, you can try uninstalling it and reinstalling an older version. See these two links for step by step explanations:

<https://stackoverflow.com/questions/3819449/how-to-uninstall-python-2-7-on-a-mac-os-x-10-6-4>

<https://apple.stackexchange.com/questions/237430/how-to-install-specific-version-of-python-on-os-x>

1. Upgrade pip

Older versions of pip have caused installation issues. If the python modules installation is difficult, try upgrading your pip:

pip3 install --upgrade pip

1. scikit-bio dependencies

scikit-bio requires specific versions of other python modules. In one of our test, installation was successful after uninstalling pandas, scipy and numpy and leaving scikit-bio take care of installing its necessary versions. First check if you have pandas, scipy and numpy already installed:

pip3 list

Then uninstall the packages:

pip3 uninstall pandas scipy numpy

And install scikit-bio only:

pip3 install scikit-bio

**Basic command**

You may use figure3.gff as a test file for the basic command:

python path/to/CRISPRStudio_1.0.py -i path/to/figure3.gff

Table S1 lists all the test files and how to use them.

**Additional parameters**

-h help message

-i GFF3 file generated with CRISPRDetect (compulsory)

-l generate the figure with a subset of isolates listed in the file (optional: by default, will generate a figure with all the strains. You need to provide a txt file with the list of the sample names exactly as they appear in the CRISPRDetect output file)

-gU gray out **unique** spacers, so that only spacers present twice in the dataset remain colored (optional, by default will attribute a unique color for each cluster of spacer)

-gS gray out **similar** spacers, so that only unique spacers remain colored (optional, by default will attribute a unique color for each cluster of spacer)

-f verification of the fasta file generated from the GFF file will be skipped if this flag is provided (optional, by default, the verification is mainly based on the length of the spacer sequences. If a spacer is 1.5 time shorter or longer than the average spacer size of the dataset, a warning is raised, and the script stops). Correction can be either made in the initial gff file or in the fasta file. If the latter, provide the name of the fasta file after the flag (ex. -f corrected_sequences.fasta)

-s sorting of the isolates in the figure: Available options: CRISPRDetect, DistMatrix or File providing a list of the isolates in the desired order. CRISPRDetect = order in the gff file. DistMatrix = Order extracted from a distance matrix based on the similarity of the arrays in the isolates. File = Order provided in a single column file with the isolates names as the should appear in the figure. The option -l overrides this option (Default: DistMatrix)

-r use this option to keep the same color attributed to the spacer during a previous analysis (optional, by default, will attribute new random colors each time the command is executed). This option may be useful when appending a preexisting dataset with new sequences if you want to have reproducible results. Run your new sequences with CRISPRDetect and copy the result at the end of your initial CRISPRDetect gff file, without changing the name of your initial file. When new sequences are added, they are aligned and clustered with the entire dataset and random colors are assigned only to new clusters, assuming new clusters are formed.

-c score cut-off for pairing of the spacers (optional, by default = 2)

-n show the size of the spacers (nucleotide length) over the boxed diamonds. We recommend using this option only for experimental analysis of the data (optional, by default, spacer size is not shown).

**Table S1**. Description of the test files

| **Command line** | python path/to/CRISPRStudio_1.0.py -i path/to/figure2_crisprstudio.gff -c 0  or any integer after -c | Follow this website for installation of crisprviz:  <https://github.com/CRISPRlab/CRISPRviz>  Then run:  crisprviz.sh -f figure2_crisprviz.fasta -x  Finally, go to your internet browser and type:  localhost:4444 | python path/to/CRISPRStudio_1.0.py -i path/to/figure3.gff | python path/to/CRISPRStudio_1.0.py -i path/to/figure6.gff -g |
| --- | --- | --- | --- | --- |
| **Description** | **gff** file containing the *Escherichia coli* strain K12 NEB 5-alpha (NZ_CP017100.1) extracted CRISPR 1 locus and its nucleotide changes at the first position of the sixth spacer. This is the test file to compare the nucleotide mismatch cut-off, which can be modified using the -c argument | **fasta** file containing the *Escherichia coli* strain K12 NEB 5-alpha (NZ_CP017100.1) extracted CRISPR 1 locus and its nucleotide changes at the first position of the sixth spacer | **gff** file containing the extracted CRISPR 1 and CRISPR 2 loci from 74 *Salmonella* strains. | **gff** file containing the extracted CRISPR 1 locus from 7 *Streptococcus thermophilus* strains. This is the test file for the graying out option. |
| **File name** | figure2_crisprstudio.gff | figure2_crisprviz.fasta | figure3.gff | figure6.gff |
