# Supplementary figures and images for: CRISPRStudio: A User-Friendly Software for Rapid CRISPR Array Visualization
Source: Viruses. 2018 Nov 1;10(11):602. doi: 10.3390/v10110602 (PMC6267562; doi:10.3390/v10110602)

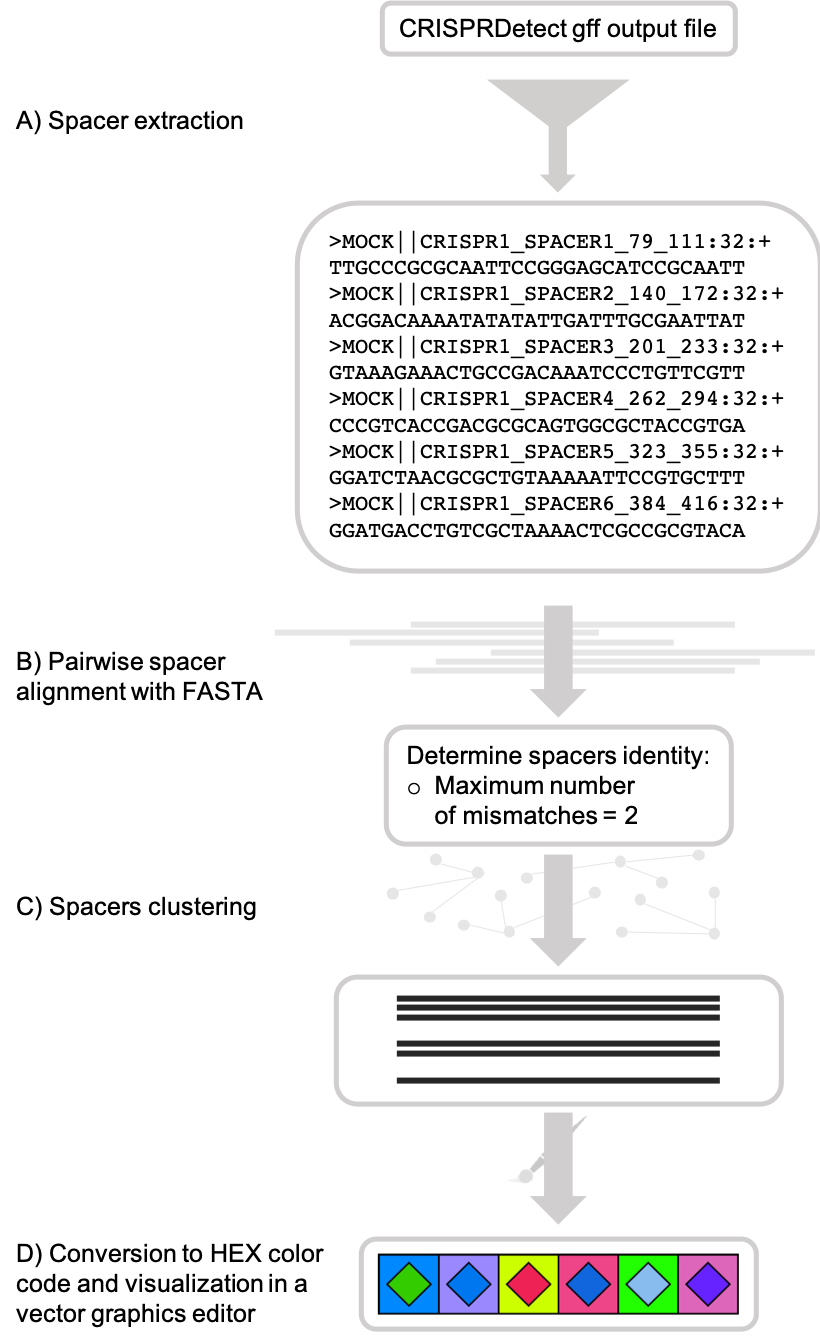

Supplement: Supplementary file 1 [file viruses-10-00602-s001.zip › 2-viruses-378352-supplrmentary/resubmission/CRISPRStudio_figures/CRISPRStudio_fig1.png]

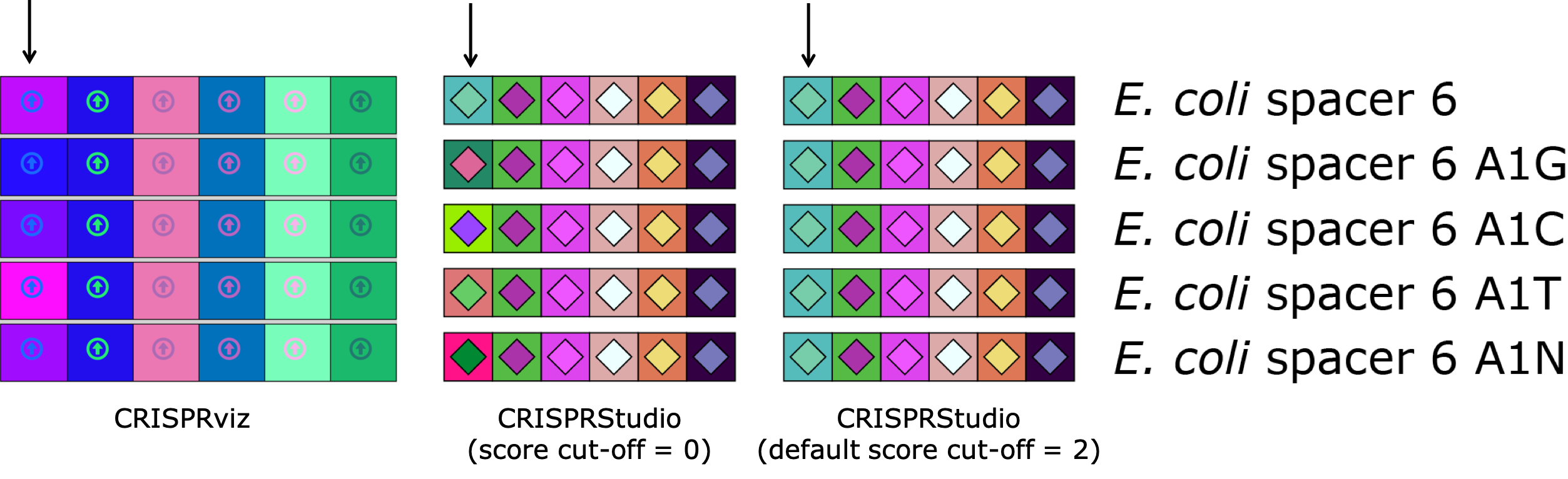

Supplement: Supplementary file 1 [file viruses-10-00602-s001.zip › 2-viruses-378352-supplrmentary/resubmission/CRISPRStudio_figures/CRISPRStudio_fig2.png]

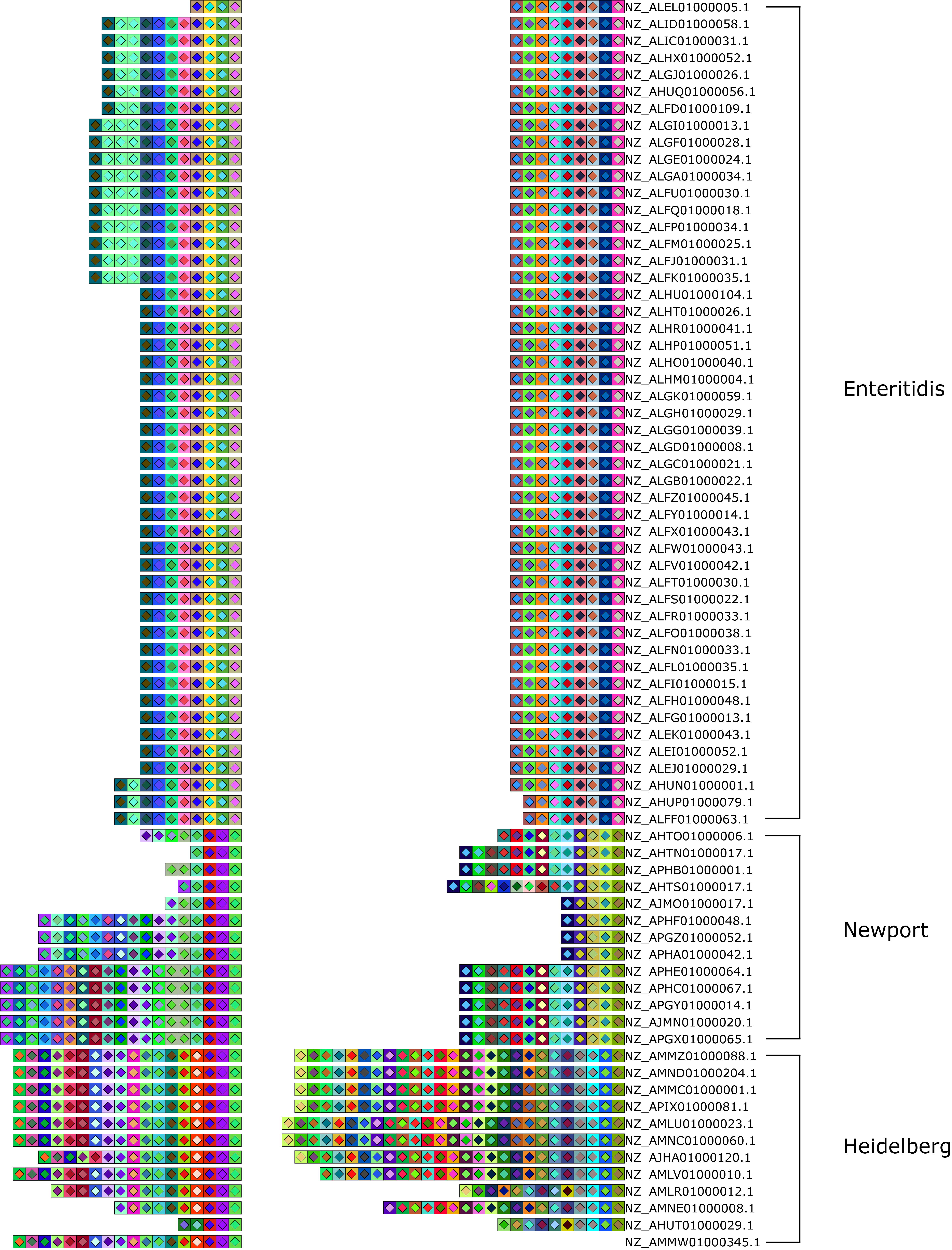

Supplement: Supplementary file 1 [file viruses-10-00602-s001.zip › 2-viruses-378352-supplrmentary/resubmission/CRISPRStudio_figures/CRISPRStudio_fig3_v2.png]

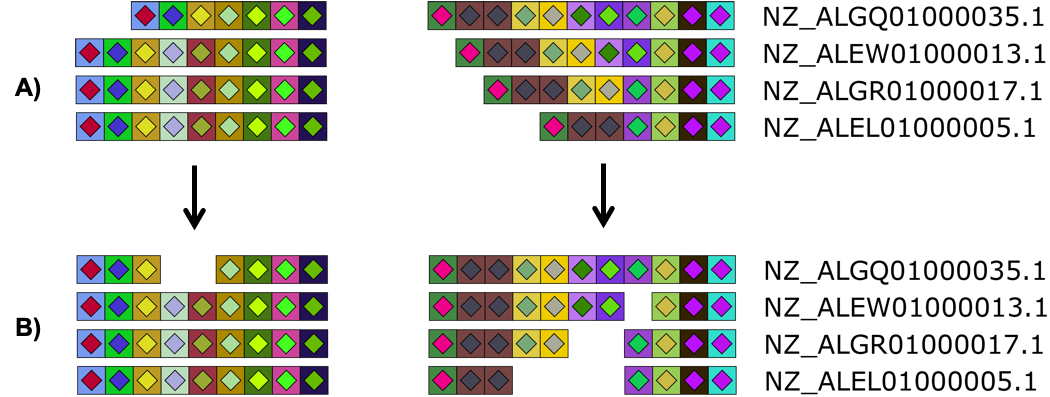

Supplement: Supplementary file 1 [file viruses-10-00602-s001.zip › 2-viruses-378352-supplrmentary/resubmission/CRISPRStudio_figures/CRISPRStudio_fig4.png]

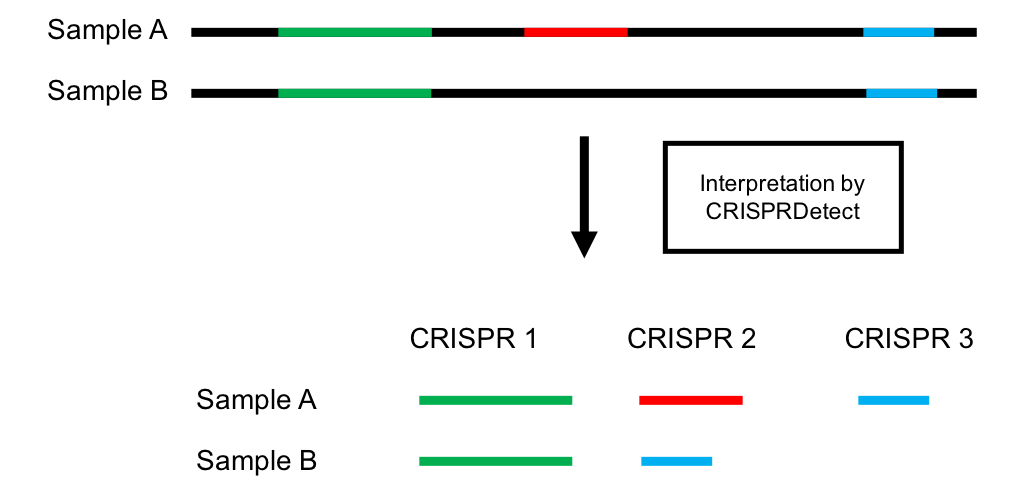

Supplement: Supplementary file 1 [file viruses-10-00602-s001.zip › 2-viruses-378352-supplrmentary/resubmission/CRISPRStudio_figures/CRISPRStudio_fig5.png]

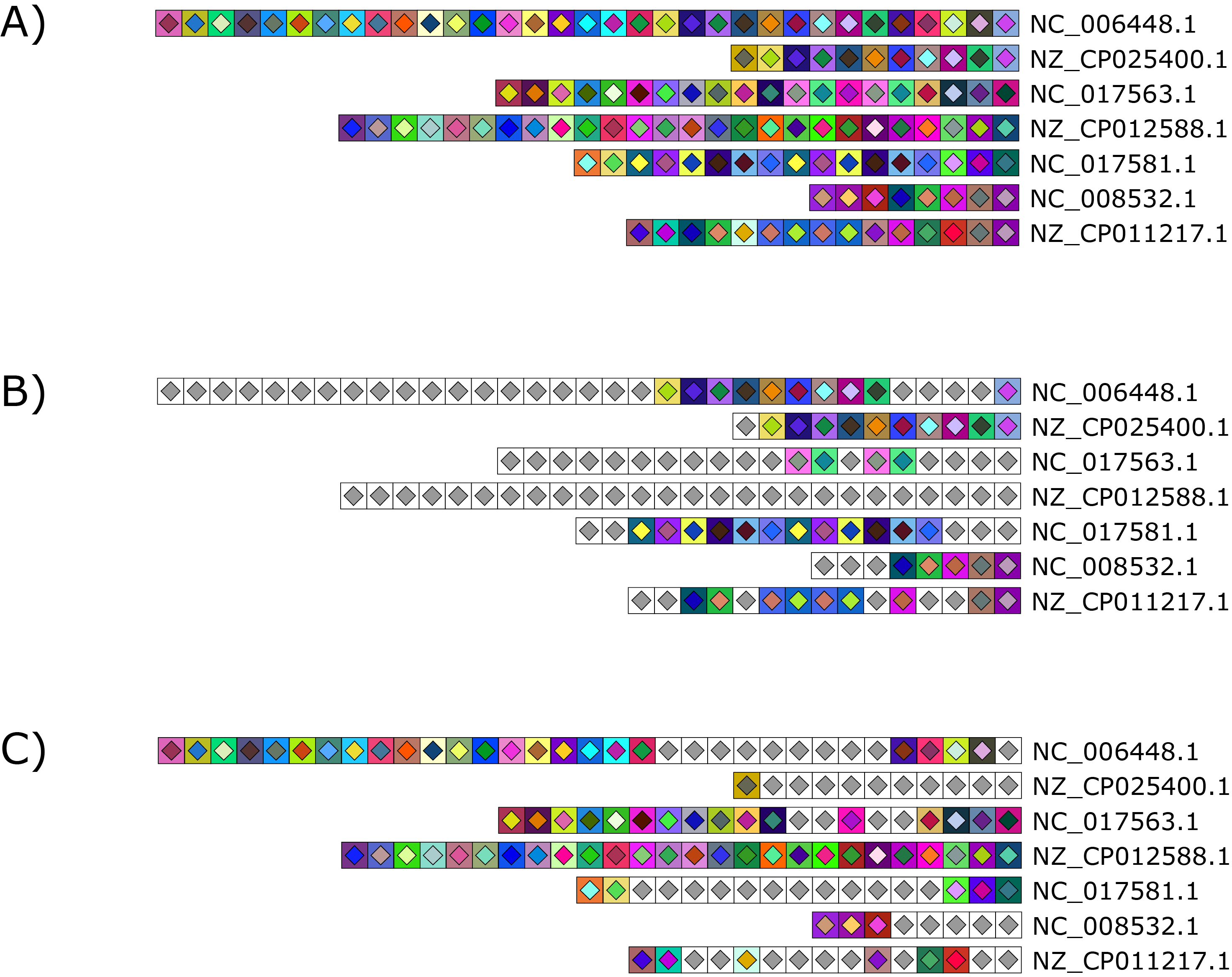

Supplement: Supplementary file 1 [file viruses-10-00602-s001.zip › 2-viruses-378352-supplrmentary/resubmission/CRISPRStudio_figures/CRISPRStudio_fig6_v2.png]

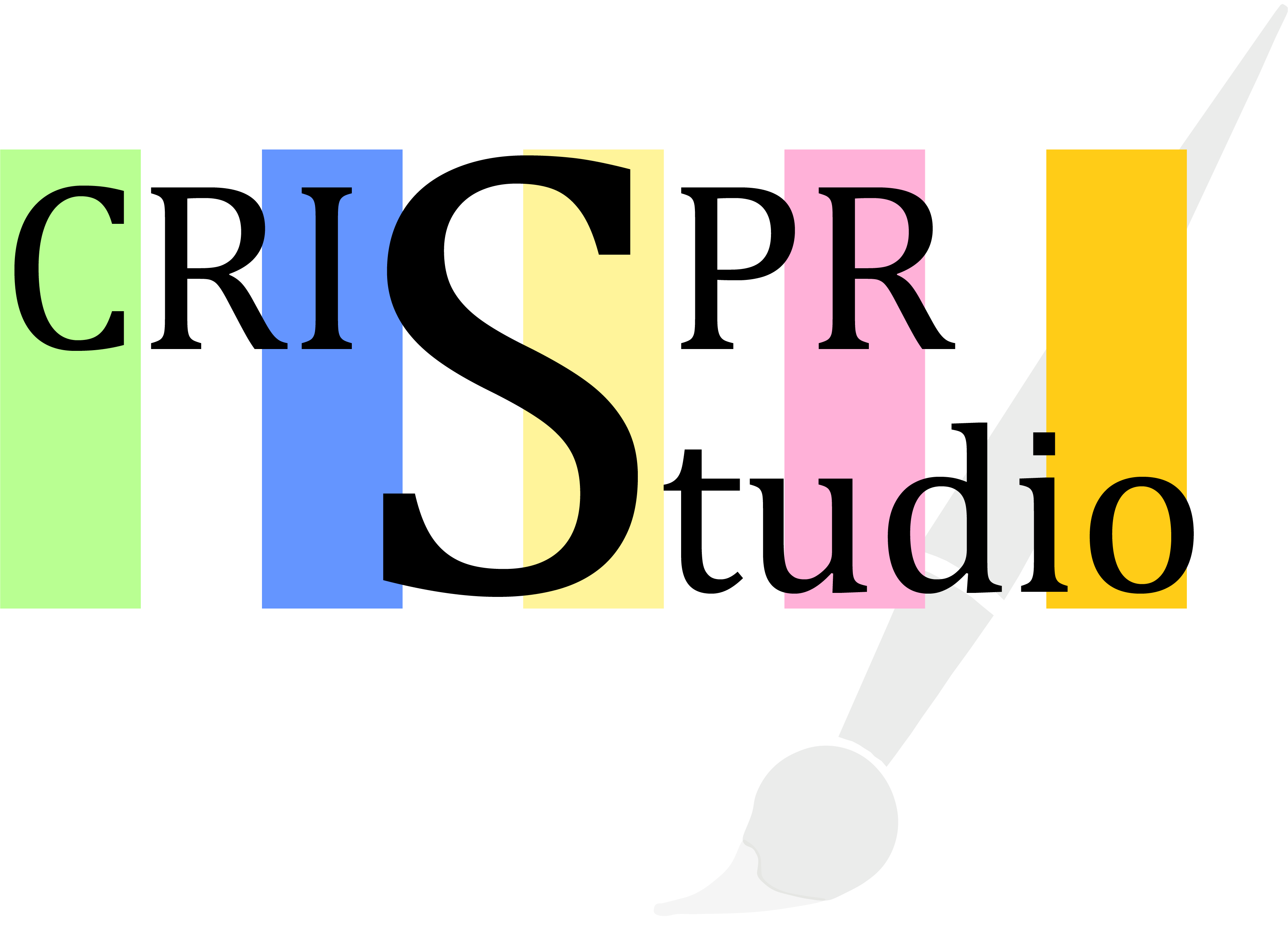

Supplement: Supplementary file 1 [file viruses-10-00602-s001.zip › 2-viruses-378352-supplrmentary/resubmission/CRISPRStudio_supp_mat/CRISPRStudio/CRISPRStudio_logo.png]
